# The RCAN1 inhibits NF-*κ*B and suppresses lymphoma growth in mice

**DOI:** 10.1038/cddis.2015.260

**Published:** 2015-10-22

**Authors:** C Liu, L Zheng, H Wang, X Ran, H Liu, X Sun

**Affiliations:** 1 Brain Research Institute, Qilu Hospital of Shandong University, 107 Wenhuaxi Road, Jinan, Shandong Province 250012, China; 2 Department of Hematology, Weifang People's Hospital, 151 Guangwen Street, Weifang 261041, China

## Abstract

Nuclear factor-*κ*B (NF-*κ*B) has a vital role in cell survival. Inhibition of NF-*κ*B has been proven to be an efficient therapeutic pathway for various cancers. Activation of NF-*κ*B is mainly through serine residues' phosphorylation of inhibitor of *κ*B*α* (I*κ*B*α*) by IKK complex. Phosphorylation at tyrosine 42 is an alternative pathway in regulation of I*κ*B*α* and NF-*κ*B signaling, though little is known about the underlying mechanism. Here we identified regulator of calcineurin 1 (RCAN1) as a novel endogenous inhibitor of NF-*κ*B signaling pathway. RCAN1 can interact with I*κ*B*α* and affect the phosphorylation of I*κ*B*α* at tyrosine 42. Overexpression of RCAN1 by adenovirus reduced cell viability in lymphoma Raji cells and restrained the growth of lymphoma transplants in mice. We further found that N terminus 1–103aa of RCAN1 is sufficient to inhibit NF-*κ*B and reduce cell viability of lymphoma cells. Our study implicated a novel therapeutic approach for lymphoma by RCAN1 through inhibition of NF-*κ*B signaling.

Nuclear factor-*κ*B (NF-*κ*B) is a family of transcription factors that share homology to the retroviral onco-protein v-Rel. NF-*κ*B is an important regulator of cell survival, proliferation and differentiation.^1^ NF-*κ*B signaling pathway has a critical role in carcinogenesis, thereby considered to be an important therapeutic target in cancer.^2^ Abnormal levels of constitutively activated NF-*κ*B have been detected in various solid tumors,^3^ leukemias, and either in cells of lymphoma patients or lymphoma cell lines.^4, 5^ NF-*κ*B could resist TRAIL-induced apoptosis in Burkitt's lymphoma (BL) cell lines infected by Epstein–Barr virus.^6^ All these results indicate that NF-*κ*B is an interesting therapeutic target in lymphoma.

The mammalian NF-*κ*B families contain five members including RelA (p65), RelB, c-Rel, p50 (p105 precursor) and p52 (p100 precursor), and share a highly conserved DNA-binding domain termed Rel homology region that enables them to form various homo- or hetero-dimeric complexes.^7^ Activation of NF-*κ*B is tightly modulated by inhibitory subunit known as inhibitor of *κ*B (I*κ*B).^8^ I*κ*B*α* binds to NF-*κ*B dimers and sterically blocks their nuclear localization sequences, thereby causing their cytoplasmic retention in rest cells. When cells are stimulated by activators, such as tumor necrosis factor *α* (TNF*α*) and lipopolysaccharide, I*κ*B*α* are phosphorylated at S32 and S36, which subsequently leads to its degradation by ubiquitin–proteasome pathway.^7^ Then NF-*κ*B dimers were released and translocated into the nucleus, where they regulate NF-*κ*B target genes' transcription. An alternative mechanism for NF-*κ*B activation involves the phosphorylation of tyrosine (Y) residue 42 of I*κ*B*α*, with or without inducing its degradation.^9, 10^ Though there were extensive studies in the serine phosphorylation by IKK complex, little is known about the regulation of I*κ*B*α* Y42 phosphorylation that also affects NF-*κ*B signaling.

The regulator of calcineurin 1 (RCAN1) is located on chromosome 21 in the region of 21q22.12. RCAN1 inhibits calcineurin–nuclear factor of activated T-cell (NFAT) pathway *in vitro* and *in vivo* by interacting with calcineurin subunit A via its C-terminal 140–197aa.^11^ Conversely, NFAT can activate RCAN1 isoform 4 gene expression via several responsive elements in the promoter region of −350–166 bp,^12, 13^ thus forming a negative feedback loop. Recent studies demonstrated involvement of RCAN1 in cancer. RCAN1 attenuates endothelial cell proliferation and angiogenesis.^13^ In the transgenic mouse model of xenografted tumors, overexpressing RCAN1 gives rise to a broad cancer protection via suppressing tumor angiogenesis by inhibiting the calcineurin pathway in the vascular endothelium.^14^ A single extra copy of RCAN1 is sufficient to suppress tumor angiogenesis in mice.^15^ Coincidently, the incidence of many cancer types, such as solid tumors, is obviously reduced in Down's syndrome patients.^16^ Our recent reports showed overexpression of RCAN1 induced neuronal apoptosis.^17^ Though we recently showed NF-*κ*B signaling regulated *RCAN1* isoform 4 gene transcription through a NF-*κ*B responsive element in the region of −576–554 bp,^18^ it is unknown if RCAN1 can conversely affect NF-*κ*B signaling activity. We were interested to know if RCAN1 can affect NF-*κ*B signaling and subsequent tumorigenesis.

In the present study, we have solid data to show that RCAN1 inhibited NF-*κ*B signaling activity. The underlying mechanism is that RCAN1 interacted with I*κ*B*α* and affected its Y42 phosphorylation. Overexpression of RCAN1 by adenovirus *in vitro* markedly reduced lymphoma Raji cell viability via activation of caspases. Overexpression of RCAN1 by adenovirus *in vivo* suppressed xenografted tumor growth in severe combined immunodeficiency (SCID) mouse model. The inhibition of NF-*κ*B activity was mediated by N terminus of RCAN1 (1–103aa), which is distinct from its C terminus (140–197aa) inhibition on calcineurin. Our studies provided a novel therapeutic pathway for lymphoma by activating RCAN1 to inhibit NF-*κ*B signaling.

## Results

### RCAN1 inhibited NF-*κ*B signaling pathway

**Figure 1:**
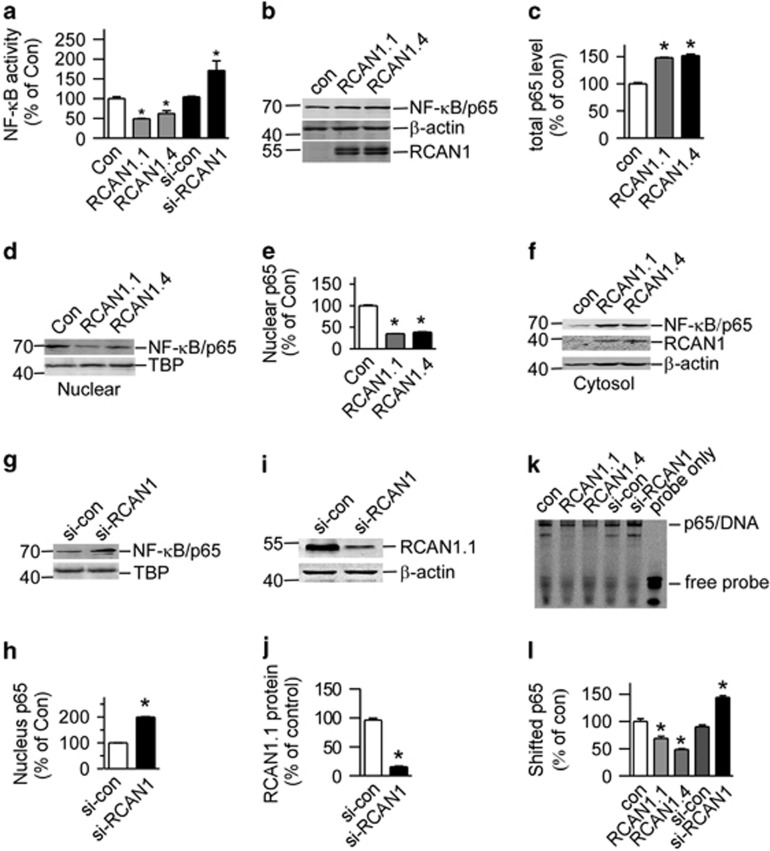
RCAN1 inhibited NF-*κ*B activity. (**a**) RCAN1 reduced the luciferase activity controlled by NF-*κ*B. RCAN1.1 and RCAN1.4 expression constructs and RCAN1 siRNA were co-transfected with pNF-*κ*BLuc into HEK293 cells. Renilla luciferase activity was used to normalize transfection efficiency. Dual luciferase assay was performed 48 h after transfection. Values represent mean±S.E.M.; *n*=4; *P<0.01 by Student's *t*-test. (**b**) Total p65 level was increased by RCAN. Plasmids pRCAN1.1/1.4-6myc were co-transfected with pNF-*κ*B into HEK293 cells. Anti-p65 antibody was used to detect the protein level of p65. Anti-myc antibody was used to detect the RCAN1 protein. *β*-actin detected by anti-*β*-actin was used as loading controls. (**c**) Quantification of **b**. Values represent mean±S.E.M.; *n*=3; *P<0.0001 by Student's *t*-test. (**d, f** and **g**) RCAN1 inhibits NF-*κ*B nuclear translocation. Nuclear proteins were extracted from HEK293 cells co-transfected with expression vectors of NF-*κ*B and pcDNA3.1RCAN1.1/1.4-mychis or si-RCAN1. NF-*κ*B/p65 protein levels were detected by anti-p65 antibody. RCAN1 protein was detected by anti-myc antibody. *β*-actin was used as loading controls for cytosol fractions. TBP detected by anti-TBP was used as loading controls for nuclear proteins. (**e** and **h**) Quantification of **d** and **g**. Values represent mean±S.E.M.; *n*=3; *P<0.0001 by Student's *t*-test. (**i**) The knockdown effect of si-RCAN1. Plasmid si-con or si-RCAN1 was co-transfected with pRCAN1.1-6myc into HEK293 cells, and anti-myc antibody was used to detect the protein level of RCAN1. *β*-actin detected by anti-*β*-actin was used as loading controls. (**j**) Quantification of **i**. Values represent mean±S.E.M.; *n*=3; *P<0.0001 by Student's *t*-test. (**k**) EMSA was performed to analyze the DNA-binding activity of NF-*κ*B. A 20-bp double-strand IRDye 800-labeled consensus NF-*κ*B oligo was used as a probe. Nuclear extracts were derived from HEK293 cells transfected with empty vector, pSi-RCAN1, pRCAN1.1mychis or pRCAN1.4-mychis. (**l**) Quantification of **h**. Values represent mean±S.E.M.; *n*=3; *P<0.001 by Student's *t*-test

To investigate whether RCAN1 has an effect on NF-*κ*B activity, RCAN1 expression plasmids pRCAN1.1mychis and pRCAN1.4-mychis were co-transfected with reporter pNF-*κ*BLuc into HEK293 cells. Dual luciferase assay showed that overexpression of RCAN1.1 and RCAN1.4 reduced NF-*κ*B luciferase reporter activity to 49.25±0.50% (*P*<0.0001) and 62.46±4.03% (*P*=0.0017) relative to controls, respectively, (lanes 2 and 3 compared with lane 1 of Figure 1a). In the si-RCAN1 (pSuper-RCAN1) transfected cells, luciferase assay showed that NF-*κ*B activity was significantly increased to 171.4±14.28% of si-control (lane 5 compared with lane 4 of Figure 1a, *P*=0.0095).

To investigate if RCAN1 affected NF-*κ*B expression, total levels of NF-*κ*B/p65 were detected in cells overexpressing RCAN1. The results showed that RCAN1 increased total NF-*κ*B/p65 expression (Figures 1b and c), precluding its effect on total levels of NF-*κ*B. NF-*κ*B activity is mainly controlled by its nuclear translocation. To examine if RCAN1 affected NF-*κ*B translocation, nuclear proteins were extracted, and NF-*κ*B/p65 western blot showed that RCAN1.1 and RCAN1.4 significantly reduced the nuclear NF-*κ*B/p65 to 34.91±0.28% and 38.4±1.16%, respectively, compared with controls (lanes 2 and 3 compared with lane 1 of Figures 1d–f, *P*<0.0001). The increase of NF-*κ*B/p65 in cytosol corresponded to the decrease in nucleus fraction, indicating the decrease of NF-*κ*B translocation in RCAN1 transfected cells.

To further confirm the effect of RCAN1 in NF-*κ*B signaling, a pSuper-based shRNA plasmid (si-RCAN1) was used to knockdown expression of RCAN1 (Figures 1g and h). Knockdown of RCAN1 increased NF-*κ*B/p65 levels in the nucleus by 199.6±1.28% (Figures 1i and j, *P*<0.0001).

To further determine the DNA-binding activity of NF-*κ*B affected by RCAN1, electrophoretic mobility shift assay (EMSA) was performed using a 20-bp double-strand IRDye 800-labeled consensus NF-*κ*B/p65 oligonucleotide (Lincoln, NE, USA) as the probe. As shown in Figure 1k, the DNA-binding activity of NF-*κ*B was elevated in cells with RCAN1 knockdown (144.3±0.03% relative to control) and reduced in cells transfected with RCAN1.1 and RCAN1.4 (69.03±0.04% and 48.53 ±0.02%, respectively, relative to control) (Figures 1k and l). These results clearly indicate that RCAN1 can inhibit the transcriptional activity of NF-*κ*B.

### RCAN1 increased the endogenous I*κ*B*α* levels

**Figure 2:**
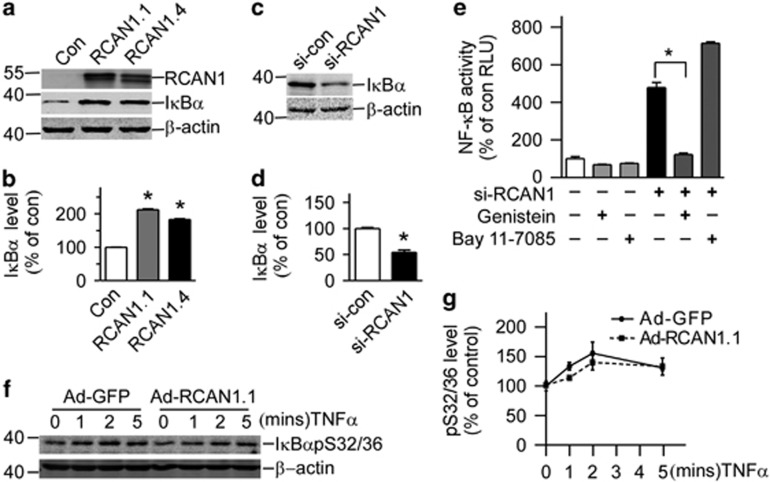
RCAN1 increased the endogenous I*κ*B*α* levels **(a**) RCAN1 increases the level of I*κ*B*α*. HEK293 cells were transfected with pcDNA3.1RCAN1.1/1.4-6myc. Endogenous I*κ*B*α* levels were detected by anti-I*κ*B*α* antibody. RCAN1 protein was detected by anti-myc antibody. *β*-actin detected by anti-*β*-actin was used as loading controls. (**b**) Quantification of **a**. Values represent mean±S.E.M.; *n*=3; *P<0.0001 by Student's *t*-test. (**c**) HEK293 cells were transfected with si-RCAN1; endogenous I*κ*B*α* levels were detected by anti-I*κ*B*α* antibody. (**d**) Quantification of **c**. Values represent mean±S.E.M.; *n*=3; *P<0.001 by Student's *t*-test. (**e**) Tyrosine kinase inhibitor inhibited NF-*κ*B activity induced by RCAN1 siRNA. HEK293 cells transfected with RCAN1 siRNA were exposed to 80 *μ*M genistein for 2 h or 5 *μ*M IKK inhibitor BAY 11-7085 for 1 h. Values represent mean±S.E.M.; *n*=4;*P<0.0001 by Student's *t*-test. (**f**) About 20 ng/ml TNF-*α* was used to treat Raji cells infected with RCAN1 adenovirus for 0, 1, 2 and 5 min. I*κ*B*α*-S32/36 phosphorylation was detected by S32/36 phosphorylation-specific antibody in western blot. *β*-actin was used as loading controls. (**g**) Quantification of **g**

As the activity of NF-*κ*B signaling was mainly regulated by IKB, endogenous I*κ*B*α* levels were detected in cells overexpressing RCAN1. The plasmids pRCAN1.1-6myc and pRCAN1.4-6myc were transfected into HEK293 cells. Western blot showed that endogenous I*κ*B*α* levels were increased to 212.2±2.19% and 183.2±2.20% in cells overexpressing RCAN1.1 and RCAN1.4, respectively (Figures 2a and b, *P*<0.0001), while knockdown of RCAN1 decreased I*κ*B*α* level to 55.44±4.19% (Figures 2c and d, *P*=0.0006).

Both the serine phosphorylation at 32 and 36 residues (mainly phosphorylated by IKK) and tyrosine phosphorylation at residue 42 will affect the degradation of I*κ*B*α* and NF-*κ*B activity.^7, 9^ To further examine which phosphorylation sites were involved, IKK inhibitor Bay 11-7085 and tyrosine kinase inhibitor Genistein were added to HEK293 cells transfected with si-RCAN1 and pNF-*κ*Bluc. Figure 2e clearly showed that inhibition of tyrosine phosphorylation by Genistein reduced NF-*κ*B activity (121.9±12.88% compared with 478.6±46.66%, *P*=0.0002, Figure 2e, lane 5 compared with lane 4), while inhibition of serine phosphorylation by IKK inhibitor Bay 11-7085 did not (Figure 2e, lane 6 compared with lane 4). To further exclude the effects on S32/36 phosphorylation, Raji cells were treated with TNF*α* to induce phosphorylation of I*κ*B*α* at Ser32/36. I*κ*B*α*-S32/36 phosphorylation antibody showed increased I*κ*B*α*-S32/36 phosphorylation after TNF*α* treatment; however, RCAN1 expression did not affect the I*κ*B*α*-S32/36 phosphorylation (Figures 2f and g). These results suggest that RCAN1 affected NF-*κ*B activity by affecting the tyrosine phosphorylation of I*κ*B*α*, which subsequently affected the NF-*κ*B activity.

### RCAN1 effected on phosphorylation of I*κ*B*α* at tyrosine 42

**Figure 3:**
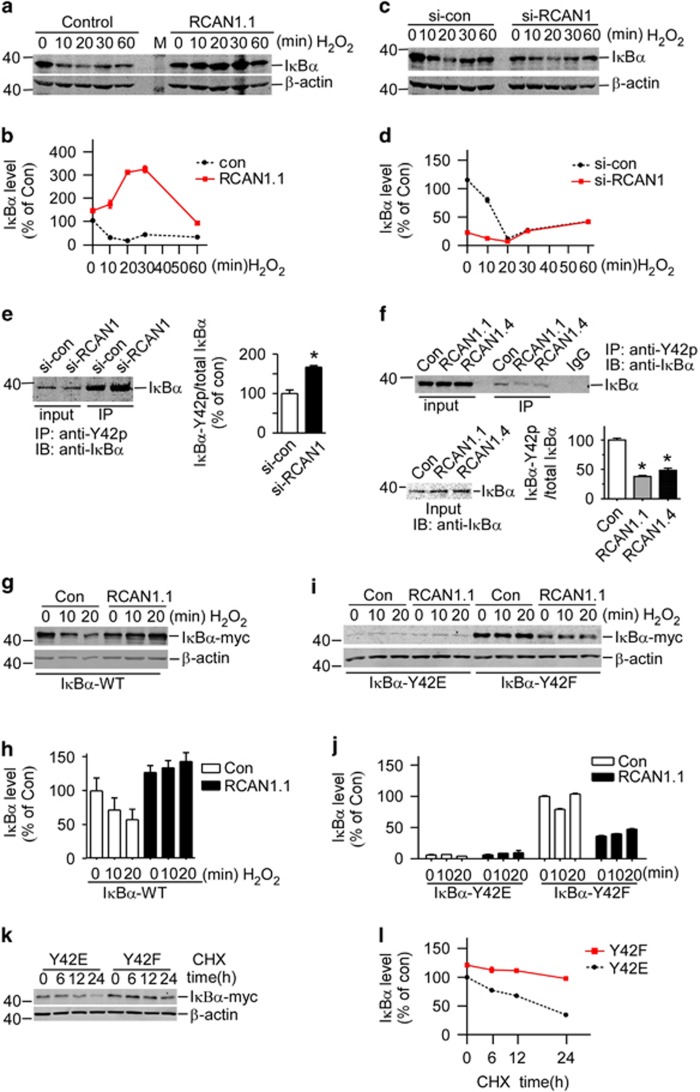
Knockdown of RCAN1 induced phosphorylation of I*κ*B*α* at tyrosine 42. (**a** and **c**) RCAN1 inhibit H_2_O_2_-induced degradation of I*κ*B*α*. RCAN1mychis and si-RCAN1 were transfected into HEK293 cells and 200 *μ*M H_2_O_2_ was added to cell cultures 48 h after transfection. Cells were harvested at each time point and endogenous I*κ*B*α* level was detected using western blot by anti-I*κ*B*α* antibody. *β*-actin was used as loading controls. (**b** and **d**) Quantification of **a** and **c**. *P*<0.0001 by two-way ANOVA. HEK293 cells were transfected with RCAN1 knockdown (**e**) or expression plasmids (**f**). The I*κ*B*α* phospho-Y42 antibody (Bioworld Tech, Cat: BS4736) was used to IP and anti-I*κ*B*α* was used in immunoblotting. The *y*-axis indicates the ratio of phosphorylated I*κ*B*α* to total I*κ*B*α*. Values represent mean±S.E.M.; *n*=3; *P<0.001 by Student's *t*-test. (**g**) RCAN1 has no effects on H_2_O_2_-induced I*κ*B*α* degradation when tyrosine 42 was mutated. I*κ*B*α* mutants Y42E and Y42F were co-transfected with pRCAN1.1mychis, and cells were exposed to H_2_O_2_ 48 h after transfection. Cells were harvested at each time point and I*κ*B*α* level was detected using western blot by anti-I*κ*B*α*. *β*-actin was used as loading controls. (**h**) Quantification of **g**. (**i**) RCAN1 inhibited H_2_O_2_ induced degradation of I*κ*B*α*-WT. HEK293 cells were co-transfected with pcDNA3.1I*κ*B*α*-WT-myc and pcDNA3.1RCAN1.1-6myc. Exogenous expressed I*κ*B*α*-WT was detected by anti-I*κ*B*α* antibody. (**j**) Quantification of **i**. (**k**) Y42 phosphorylation affected I*κ*B*α* stability. Plasmids pI*κ*B*α*-Y42E/F were transfected into HEK293 cells separately. About 48 h after transfection, the cells were exposed to 0.5 *μ*M lactacystin (lac) for 4 h, and then chased with 50 ng/ml cycloheximide (CHX) for 6, 12 and 24 h. I*κ*B*α* level was detected using western blot by anti-I*κ*B*α* antibody. *β*-actin was used as loading controls. (**l**) Quantification of **k**

It was previously reported that oxidative stress could activate NF-*κ*B through Y42 phosphorylation of I*κ*B*α* via spleen tyrosine kinase (SYK).^19, 20^ To further confirm the effect of RCAN1 on I*κ*B*α* Y42 phosphorylation, hydrogen peroxide (H_2_O_2_) was used to induce Y42 phosphorylation of I*κ*B*α*. H_2_O_2_ induced the degradation of endogenous I*κ*B*α* in a time-dependent manner (left panel of Figures 3a and b); in contrast, RCAN1 expression greatly increased I*κ*B*α* levels, particularly at 10, 20 and 30 min after H_2_O_2_ addition (right panel of Figures 3a and b). Consistent with this, RCAN1 knockdown decreased I*κ*B*α* levels after addition of H_2_O_2_ at 10 and 20 min compared with control (Figures 3c and d). The effect of H_2_O_2_ on endogenous I*κ*B*α* degradation is strictly dependent on time, in that the effect is most obvious at 20 min (Figures 3a and c).

To further confirm that the effect of RCAN1 was through the Y42 phosphorylation of I*κ*B*α*, the I*κ*B*α* phospho-Y42 antibody (Bioworld Tech, Nanjing, Jiangsu, China) was used to immunoprecipitation (IP) and anti-I*κ*B*α* was used in immunoblotting. The results showed knockdown of RCAN1 increased the ratio of Y42 phosphorylated I*κ*B*α* (Figure 3e) and RCAN1 expression decreased the Y42 phosphorylated I*κ*B*α* (Figure 3f). These results demonstrated that RCAN1 inhibited NF-*κ*B signaling by interacting with I*κ*B*α* and affecting its phosphorylation at Y42.

To further confirm the effect of RCAN1 is through the Y42 residue of I*κ*B*α*, two mutant I*κ*B*α*, I*κ*B*α*-Y42E and I*κ*B*α*-Y42F, were constructed and co-transfected with RCAN1. Compared with the increase of I*κ*B*α*-WT by RCAN1 expression after addition of H_2_O_2_ (Figures 3g and h), mutation of Y42 residue abolished the increasing effects of RCAN1 on I*κ*B*α* (Figures 3i and j). The weak bands in I*κ*B*α*-Y42E indicated a faster degradation compared with I*κ*B*α*-Y42F (Figures 3i and j), suggesting that the Y42 phosphorylation can lead to its degradation. Proteasome inhibitor lactacystin ((lac) was used to stabilize and visualize the I*κ*B*α*-Y42E expression. Plasmids pI*κ*B*α*-Y42E/F were transfected into HEK293 cells and 0.5 *μ*M lac was added to the cells after 48 h transfection. About 4 h after lac was added, the cells were chased with 50 ng/ml cycloheximide. I*κ*B*α*-Y42E turnover rate was much faster than the Y42F mutant (Figures 3k and l), suggesting the Y42 phosphorylation indeed affected the I*κ*B*α* stability. These results demonstrated that RCAN1 could affect Y42 phosphorylation and subsequently I*κ*B*α* protein stability.

### RCAN1 decreased cell viability in lymphoma Raji cell lines

**Figure 4:**
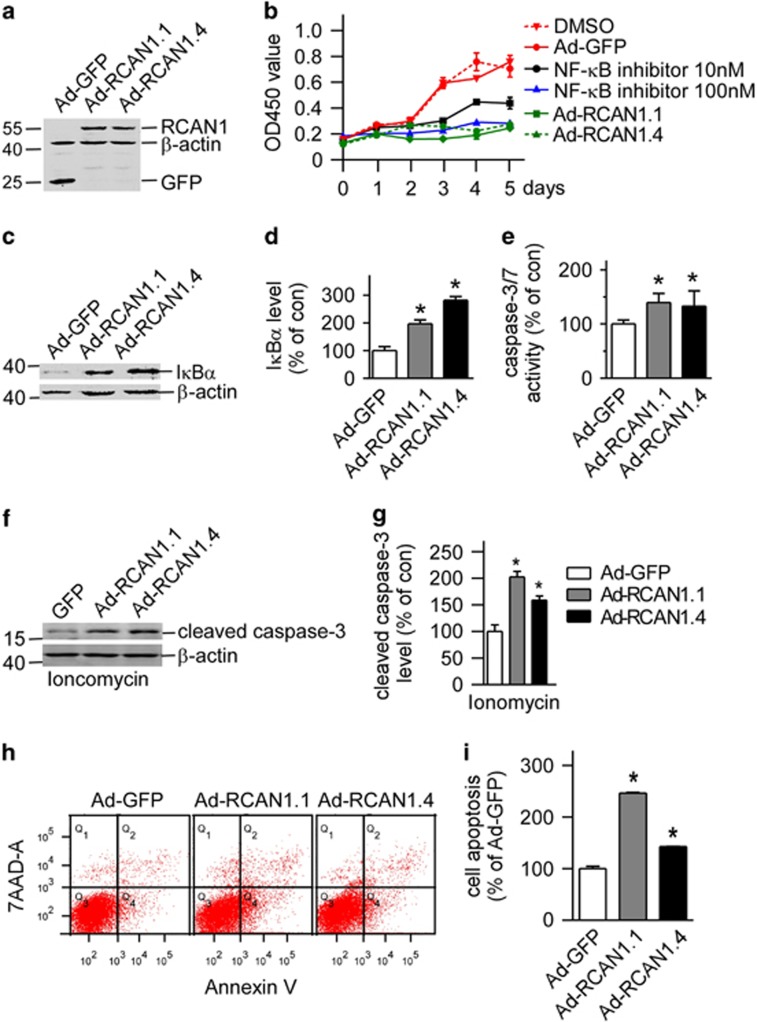
Overexpression of RCAN1 decreased lymphoma Raji cell viability (**a**) Expression of RCAN1 isoform 1 and 4 in Raji cells by RCAN1.1 and RCAN1.4 adenovirus. Anti-GFP antibody was used to detect the expression of RCAN1. *β*-actin was used as loading controls. (**b**) RCAN1 reduced Raji cell viability. Raji cells infected with adenovirus overexpressing RCAN1.10 and 100 nM NF-*κ*B activation inhibitor (Calbiochem, #481406) were used as positive controls. Dimethyl sulfoxide (DMSO) was the control for NF-*κ*B inhibitor. Ad-GFP infected cells was used as negative control for RCAN1. CCK8 cell viability was measured by the absorbance at 450 nm (ref: 650 nm) at indicated times after adenovirus infection. *P*<0.0001 by two-way ANOVA. (**c**) Anti-I*κ*B*α* was used to detect endogenous I*κ*B*α* protein level in Raji cells infected with RCAN1 adenovirus. *β*-actin was used as loading controls. (**d**) Quantification of **c**. Values represent mean±S.E.M.; *n*=3; *P<0.001 by Student's *t*-test. (**e**) Raji cells were infected with Ad-GFP, Ad-RCAN1.1-GFP and Ad-RCAN1.4-GFP. Caspase-3/7 activity was measured 48 h after infection. Values represent mean±S.E.M.; *n*=3; *P<0.05, by Student's *t*-test. (**f**) Anti-cleaved caspase-3 (Asp175 from CST) was used to detect the cleaved caspase-3 protein level in Raji cells that were infected with RCAN1 adenovirus and treated with 5 *μ*M ionomycin. *β*-actin was used as loading controls. (**g**) Quantification of **f**. Values represent mean±S.E.M.; *n*=3; *P<0.001 by Student's *t*-test. (**h**) Raji cells infected with RCAN1 adenovirus were stained with 7-AAD and Annexin V and analyzed by FACS to detect cell apoptosis. (**I**) Quantification of **h**. Values represent mean±S.E.M.; *n*=3; *P<0.001 by Student's *t*-test

NF-*κ*B is the major survival factor in cells and constitutively activated NF-*κ*B has been observed in various lymphomas. To investigate if the NF-*κ*B inhibition by RCAN1 will affect cell viability, lymphoma Raji cells were infected with adenovirus expressing RCAN1.1 and RCAN1.4 (Figure 4a). Ad-RCAN1.1 and Ad-RCAN1.4 markedly decreased cell viability in 3, 4 and 5 days after adenovirus infection (Figure 4b, *P*<0.0001). The potent NF-*κ*B activation inhibitor (Calbiochem, #481412, Beijing, China) was used as positive controls (Figure 4b, *P*<0.0001). The effects of RCAN1 overexpression on cell viability were comparable to 100 nM NF-*κ*B activation inhibitor and much better than 10 nM inhibitor. Concurrently, RCAN1.1 and RCAN1.4 increased the level of I*κ*B*α* in Raji cells to 196.9±8.14% (*P*=0.0011) and 282.0±7.46% (*P*<0.0001), respectively (Figures 4c and d). The decrease of cell viability detected by Cell Counting Kit-8 (CCK8) can be either due to increased cell death or cell cycle arrest. The flow cytometry test of cell cycle showed RCAN1 had no effect on cell cycle. We previously showed RCAN1 expression induced neuronal apoptosis. To elucidate if RCAN1 can induce Raji cell apoptosis, caspase-3/7 activity was examined in Raji cells infected with RCAN1 adenovirus. The caspase-3/7 activity assay showed RCAN1 increased caspase-3/7 activity in Raji cells (Figure 4e, *P*<0.01). Activation of caspase-3 can also be indicated by its cleavage from 32 to 17 kD. RCAN1.1 and RCAN1.4 increased the level of cleaved caspase-3 in Raji cells to 202.24±6.08% (*P*=0.0004) and 159.2±4.36% (*P*=0.0021), respectively, (Figures 4f and g). Furthermore, the flow cytometry of Annexin V also showed that RCAN1 increased Raji cell apoptosis to 246.6±1.19% (*P*<0.0001) and 142.5±0.78% (*P*=0.0008) (Figures 4h and i). These data suggest that RCAN1 induced the apoptosis of Raji cells via inhibiting the NF-*κ*B signaling pathway.

### RCAN1 suppressed the growth of lymphoma xenografts in SCID mice

**Figure 5:**
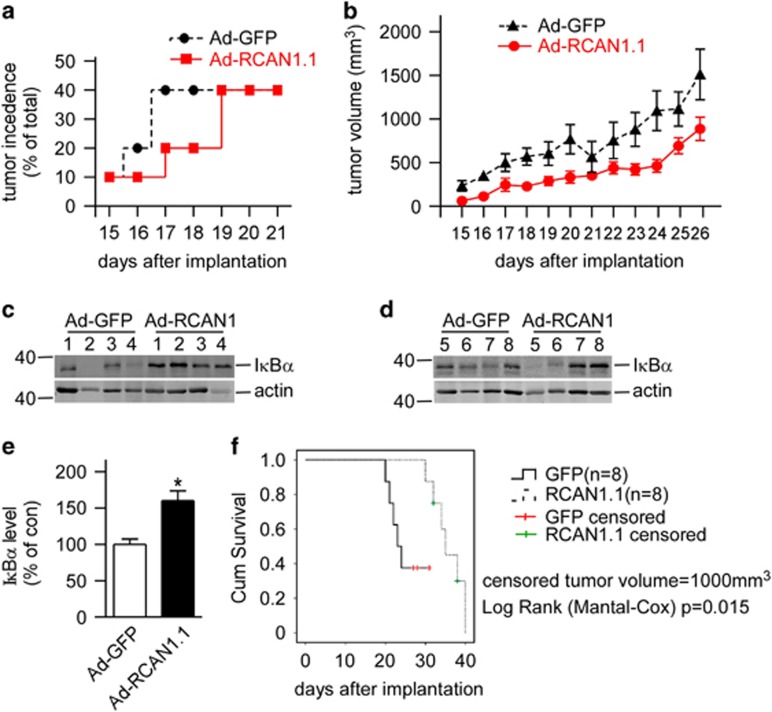
Overexpression of RCAN1 suppressed the growth of lymphoma xenograft tumor in SCID mice. (**a**) RCAN1 decreased incidence of lymphoma xenograft in SCID mice. The *x*-axis indicated the days after transplantation. (**b**) The tumor volume was calculated from the tumor diagram measured every day. The *x*-axis indicated the days after transplantation. *P*=0.0008 by two-way ANOVA. (**c**) The mice were killed and tumors were dissected. The tumor tissues were homogenated and separated with SDS-PAGE. Anti-I*κ*B*α* antibody was used to detect endogenous I*κ*B*α* and *β*-actin was used as loading controls. (**d**) Quantification of **c**. Values represent mean±S.E.M.; *n*=8; *P=0.0013 by Student's *t*-test. (**e**) The tumor volume of 1000 mm^3^ was defined as the censored event in the log rank Mantel–Cox test (*P*=0.015), which was analyzed with SPSS17.0 statistics software (Armonk, NY, USA)

To further confirm that RCAN1 inhibited NF-*κ*B signaling *in vivo*, Raji cells infected with RCAN1.1 or GFP adenovirus were s.c. injected into SCID mice. Lymphoma formation was observed ~2 weeks after Raji cells transplantation. The tumor occurrence time was delayed in the RCAN1.1 group compared with GFP control group (Figure 5a). Furthermore, the tumor volumes were also smaller in RCAN1.1 group compared with GFP controls (Figure 5b, *P*=0.0008). About 80% (8/10) of mice for each group had tumors formed after 38 days post transplantation. The experiment was ended 42 days after transplantation. The tumors were dissected and the endogenous I*κ*B*α* levels were examined in tumor homogenates. Western blot using the tumor tissues showed I*κ*B*α* was significantly elevated in the RCAN1.1 group (Figures 5c and d, *P*=0.0013). The log rank test showed that the tumor growth in RCAN1 overexpressed group is slower than the control (Figure 5e, *P*=0.015). The results indicated that RCAN1 reduced the incidence, as well as the growth of lymphoma xenografts in mice.

### RCAN1 N-terminal domain 1–103aa directly interacted with I*κ*B*α*

**Figure 6:**
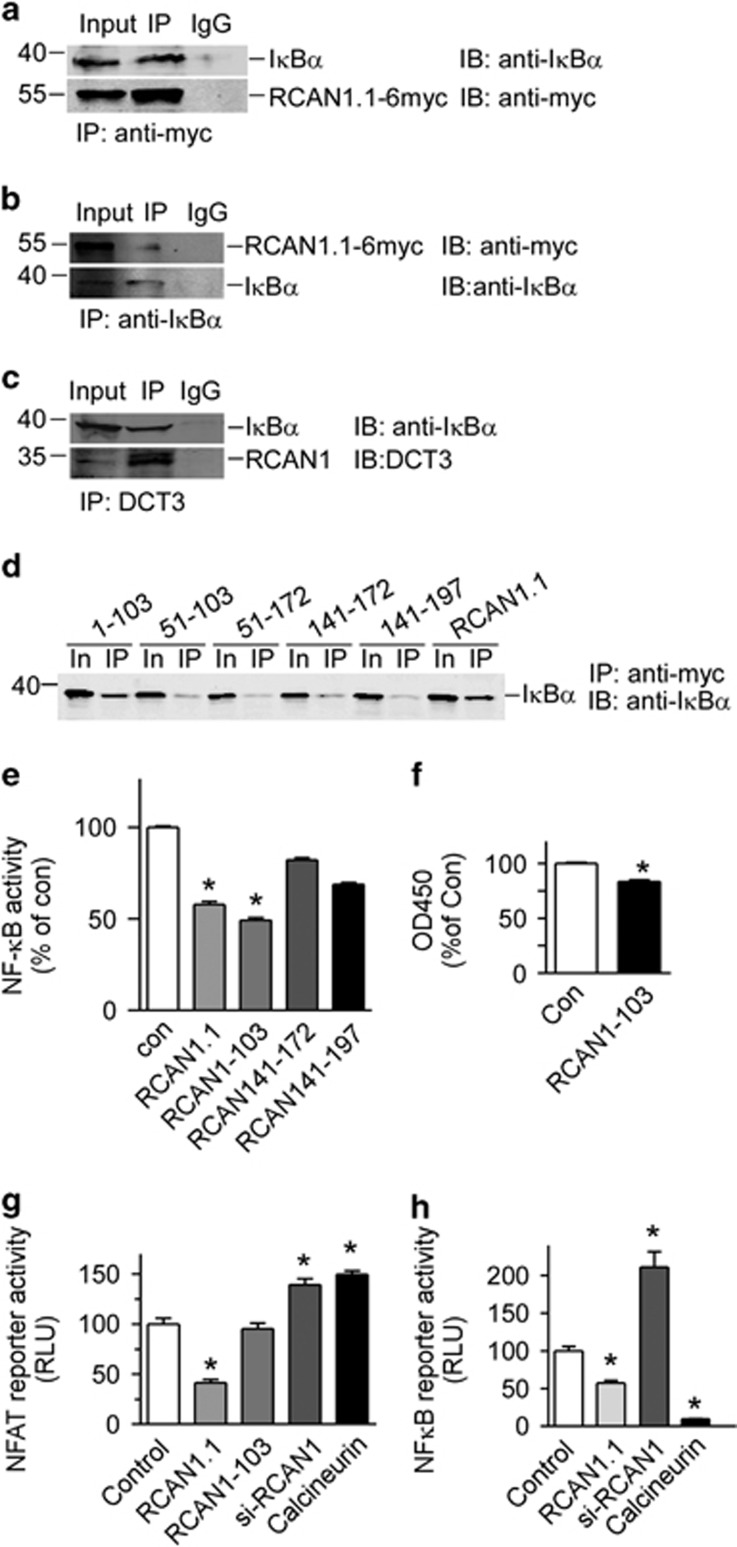
RCAN1 directly interacted with I*κ*B*α* through its N-terminal 1–103aa. (**a** and **b**) RCAN1 interacted with I*κ*B*α*. Co-IP was performed using HEK293 cells transfected with pRCAN1.1-6myc. 9E10 was used as the pull-down antibody and anti-I*κ*B*α* antibody was used as detection antibody in **a**, and *vice versa* in **b**. (**c**) Endogenous RCAN1 interacted with I*κ*B*α*. Co-IP was performed using HEK293 cells. DCT3 antibody targeting RCAN1 C terminus was used as the pull-down antibody and anti-I*κ*B*α* antibody was used as detection antibody. (**d**) RCAN1.1 deletion mutants were transfected into HEK293 cells. Anti-myc beads (Sigma, E6654, Shanghai, China) were used as IP and anti-I*κ*B*α* was used in western blot. The numbers above indicate the fragments of RCAN1.1. In, input. The antibody 9E10 was used to detect the expression of RCAN1 mutants. (**e**) RCAN1-103 inhibited the transcriptional activity of NF-*κ*B. Plasmids expressing RCAN1.1, RCAN1-103, RCAN141-172 and RCAN141-197 were co-transfected with pNF-*κ*BLuc into HEK293 cells. Renilla luciferase activity was used to normalize transfection efficiency. Dual luciferase assay was performed 48 h after transfection. Values represent mean±S.E.M.; *n*=3; *P<0.0001 by Student's *t*-test. (**f**) RCAN1-103 reduced the Raji cell viability. Cells were infected with adenovirus expressing RCAN1 1–103aa. Cell viability assay was performed 4 days after infection. Values from GFP control were arbitrarily set to 100%. Values represent mean±S.E.M.; *n*=4; *P<0.0001 by Student's *t*-test. (**g** and **h**) Inhibition of NF-*κ*B by RCAN1 is independent of its inhibition on calcineurin. The plasmids RCAN1-103 containing 1–103aa from N terminus of RCAN1.1, expression vector of calcineurin, as well as pRCAN1.1-6myc and pSi-RCAN1 were co-transfected with pNFATluc (**g**) or pNF-*κ*Bluc (**h**) reporter plasmid. Dual luciferase assay was performed 48 h after transfection. Values represent mean±S.E.M.; *n*=3; *P<0.01, by Student's *t*-test

To investigate if there is an interaction between RCAN1 and I*κ*B*α*, a co-IP assay was performed. HEK293 cells were transfected with pRCAN1.1-6myc. Anti-myc (9E10) was used to pull down RCAN1. Our results showed that anti-I*κ*B*α* detected a band in 9E10 IPs that migrated together with I*κ*B*α* protein in the cell lysate (Figure 6a). And, *vice versa*, the 9E10 antibody detected RCAN1 protein in the anti-I*κ*B*α* antibody pull down (Figure 6b). The endogenous RCAN1 and I*κ*B*α* interaction was also confirmed by Co-IP using DCT3 antibody,^17^ targeting the RCAN1 C terminus (Figure 6c). These results indicated that there is a direct interaction between RCAN1 and I*κ*B*α* proteins.

To further elucidate the domain of RCAN1 interacting with I*κ*B*α*, a series of deletion mutants was constructed. Co-IP assay showed that only 1–103aa from N terminus of RCAN1.1 can interact with I*κ*B*α*, while 51–103aa, 51–172aa, 141–172aa and 141–197aa did not interact with I*κ*B*α* (Figure 6d). Luciferase assay also confirmed that the 1–103aa of RCAN1.1 inhibited NF-*κ*B activity (lane 3 of Figure 6e, *P*<0.0001), similarly with the RCAN1 full-length (lane 2 of Figure 6e). While the deletion constructs of 141–172aa and 141–197aa did not inhibit the NF-*κ*B activity (lane 4 and 5 of Figure 6e). Furthermore, the RCAN1 N terminus 1–103aa also reduced cell viability measured with CCK8 assay (83.51±0.0152% of control, Figure 6f, *P*<0.0001). These results verified that RCAN1 N terminus (1–103aa) can inhibit NF-*κ*B via its physical interaction with I*κ*B*α*.

### Inhibition of NF-*κ*B by RCAN1 was independent of its inhibition on calcineurin

RCAN1 is previously known to be an inhibitory regulator of calcineurin. Knockdown of RCAN1 would increase calcineurin activity, in that calcineurin overexpression and knockdown of RCAN1 would show similar effects if inhibition of NF-*κ*B by RCAN1 was through its regulation of calcineurin. To investigate whether the inhibition of NF-*κ*B by RCAN1 is associated with calcineurin, the calcineurin expression plasmid and si-RCAN1 were co-transfected with pNF-*κ*Bluc or pNFATluc reporter into HEK293 cells. Luciferase assay showed overexpression of calcineurin or knockdown of RCAN1 increased luciferase activity of NFATluc to 149.7±3.35 and 139.3±5.96% relative to control (lanes 4 and 5 of Figure 6g), indicating that RCAN1 regulated NFAT activity by its effect on calcineurin. However, in pNF-*κ*Bluc-transfected cells, calcineurin significantly inhibits NF-*κ*B-controlled luciferase activity to 9.51±0.495% (lane 4 of Figure 6h), while knockdown of RCAN1 increased NF-*κ*B luciferase activity to 211.2±20.2% (lane 3 of Figure 6h). The N-terminal domain of RCAN1 1–103aa can inhibit the NF-*κ*B activity (Figure 6e), while it has no effect on NFAT activity (lane 3 of Figure 6g). The opposite effect of calcineurin overexpression and RCAN1 knockdown on NF-*κ*B activity indicate that the inhibition of NF-*κ*B by RCAN1 is independent upon its inhibition of calcineurin, the former is related to the N terminus (1–103aa) and the latter is associated with its C terminus (140–197aa).^11^

## Discussion

Our study here identified RCAN1 as a novel inhibitor of NF-*κ*B signaling pathway. The underlying mechanism is that N terminus 1–103aa of RCAN1 can physically interact with I*κ*B*α* and affect its Y42 phosphorylation. The RCAN1 overexpression in cells leads to Raji cell death *in vitro*. And RCAN1 expression *in vivo* reduced tumor growth in xenografted lymphomas in SCID mice. Sustained activation of NF-*κ*B is prevalent in cell lines and tumor tissue specimens and contributes to malignant progression and therapeutic resistance in most of the major forms of human cancer.^21^ As a newly approved drug for multiple myeloma, the proteasome inhibitor bortezomib (VELCADE) can inhibit the degradation of I*κ*B*α* and subsequently inhibit NF-*κ*B signaling pathway.^22^ Since NF-*κ*B is a popular cancer drug target, the identification of RCAN1 as an NF-*κ*B inhibitor provides a potential treatment for cancers in which NF-*κ*B signaling is aberrantly activated. Our data here showed that RCAN1 affected I*κ*B*α* Y42 phosphorylation. Previous reports have shown that RCAN1 overexpression stabilized I*κ*B*α*^23^ and deficiency of RCAN1 led to increased NF-*κ*B activity.^24, 25^ Our study here further provided the molecular mechanism of RCAN1 effect on I*κ*B*α* Y42 phosphorylation. High glucose and reactive oxygen species have been shown to be able to activate NF-*κ*B signaling via I*κ*B*α* Y42 phosphorylation by spleen tyrosine kinase (SYK).^26^ Continuous studies will be needed to elucidate if RCAN1 has interactions with I*κ*B*α* tyrosine kinases such as SYK.

Single extra transgenic copy of *Dscr1* is sufficient to confer significant suppression of tumor growth in mice, and that such resistance has thought to be a consequence of a deficit in tumor angiogenesis arising from suppression of the calcineurin pathway.^14, 27^ Hence, it is reasonable to speculate that cyclosporin A and FK506 (tacrolimus), immunosuppressive drugs that specifically inhibit calcineurin,^28^ would also suppress tumor angiogenesis. Surprisingly, numerous clinical studies indicate that a significant increase in cancer incidence is a serious complication of transplant recipients receiving long-term immunosupressive therapy.^29^ The mechanism behind this increased rate of cancer is not yet understood; however, such studies indicate another oncogenic pathway other than inhibition of calcineurin in which RCAN1 may be involved. RCAN1's inhibition of NF-*κ*B is independent of its inhibition on calcineurin, thus providing a novel mechanism for tumor suppressive effect of RCAN1. Further, we found that the N terminus of RCAN1 is sufficient in inhibiting the NF-*κ*B signaling and reducing the lymphoma cell viability, while it has no effect on NFAT signaling.

RCAN1 not only can repress NFAT signaling pathway via inhibition of calcineurin,^30^ but also can be activated by activators of calcineurin–NFAT pathway such as calcium ionophore, VEGF, angiotensin II, TNF-*α* and so on,^12, 13^ thereby forming a negative feedback loop in RCAN1 gene regulation. Our recent study showed NF-*κ*B can activate RCAN1 isoform 4 gene promoter through a NF-*κ*B responsive element in the region of −576–554 bp.^18^ Our study here showed inhibition of NF-*κ*B by RCAN1, thus forming a second negative feedback loop in regulating RCAN1 gene expression by NF-*κ*B. The tight regulation of RCAN1 gene expression by negative feedback loops involving NFAT and NF-*κ*B implies the crucial role of RCAN1 in regulation of cellular functions.

## Materials and Methods

### Plasmids construction

The plasmids pRCAN1.1-6myc, pRCAN1.4-6myc, pSuper-RCAN1 and pRCAN51-172myc were generated as previously described.^31, 32, 33^ RCAN1.1 and RCAN1.4 are the two major isoforms of RCAN1, containing exons 1, 5, 6, 7 and exons 4, 5, 6, 7 respectively. RCAN1-103 was PCR amplified with T7 primer and DS103KpnR (5′-ATGGTACCGCTTGTCTGGATTTGGCGGA-3′) from pRCAN1.1mychis and cloned into pRCAN1.1-6myc to make pRCAN 1-103myc. RCAN 51-103 was PCR amplified with RCAN1-51EcorF (5′-GGAATTCCAAACGAGTCAGAATAAA-3′) and DS103KpnR from pRCAN1.1mychis and cloned into pRCAN1.1-6myc to make pRCAN 51-103myc. RCAN141-172 was PCR amplified with primer DS141XhoF (5′- CCGCTCGAGGCCACCATGGGGGAAAAGTATGAATTGC-3′) and RCAN1-KpnR from pRCAN1.1mychis and cloned into pRCAN1.1-6myc to make pRCAN 141-172myc. RCAN141-197 was PCR amplified with DS141XhoF and BGH primers from pRCAN1.1mychis and cloned into pRCAN1.1-6myc to make pRCAN 141-197myc. The fragments of RCAN1.1-GFP and RCAN1.4-GFP were subcloned into shuttle plasmid pHMCMV5 from pRCAN1.1-GFP and pRCAN1.4-GFP using Nhe1 and Not1, and then transferred to adenovirus vector pAd5F35 plasmid to generate pAd-RCAN1.1-GFP and pAd-RCAN1.4-GFP as previously described.^34^ The I*κ*B*α* mutant plasmids I*κ*B*α*-Y42E and I*κ*B*α*-Y42F were constructed by site-directed mutagenesis.

### Generation and purification of recombinant adenovirus

About 5 *μ*g *Pac I*-digested pAd-RCAN1.1 and pAd-RCAN1.4 were transfected into HEK293 cells using lipofectamine 2000 transfection reagent (Invitrogen, Waltham, MA, USA) in a 60-mm cell culture plate. Seven days later, cells were harvested and lysed with three consecutive cycles of freezing–thawing in a methanol/dry ice bath. Then 50% of the cell lysates were used to infect HEK293 cells in a 60-mm culture plate. Recombinant adenoviruses were amplified with the re-infection of HEK293 cells twice more.

For the purification of adenovirus, we collected 25 175cm^2^-culture flasks of adenovirus-affected HEK293 cells. After three freeze–thaw cycles, the supernatants were added into a 50 ml centrifuge tube with 1.33 and 1.45 M cesium chloride. After 1.5 h centrifugation at the speed of 18 000 r.p.m. at 4 °C, the adenoviruses layer was collected and dialyzed against 1 × PBS overnight at 4 °C. The titer of adenovirus was determined directly by GFP expression 48 h after transduction using the limiting dilution method. The titer for Ad-GFP, Ad-RCAN1.1-GFP and Ad- RCAN1.4-GFP was 5.00 × 10^13^, 7.94 × 10^13^ and 3.16 × 10^13^ p.f.u./ml. The lymphoma cell lines were infected with a multiplicity-of-infection of 30. The infection efficiency 48 h after infection is about 80–90% in Raji cells.

### Cell culture, cell viability and apoptosis assay

HEK293 cells was cultured as previously described.^33^ Human BL cell line Raji were cultured in RPMI 1640 (Hyclone, South Logan, UT, USA) medium supplemented with 10% FBS, 1 mM glutamine, 10 mM Hepes and 100 U/ml penicillin-streptomycin. All cells were maintained at 37 °C in an incubator containing 5% CO2.

Cell viability was detected with CCK8 (Cat: #C0037, Beyotime, Shanghai, China) according to the manufacturer's instructions. Briefly, 10 000 cells were seeded in 96-well cell culture plate and infected with Ad-GFP or Ad-RCAN1.1-GFP for 4 days. Four hours after CCK8 was added, absorbance at 450 nm (ref: 650 nm) was detected with the Thermo Scientific microplate reader Varioskan Flash (Waltham, MA, USA). Caspase-3/7 activity was measured using the Caspase-Glo 3/7 assay kit (G8090, Promega, Madison, WI, USA) according to the manufacturer's instructions as previously described.^32^ The flow cytometry was performed using 7-AAD and Annexin staining kit (eBioscience, Cat: #88-8102), detected by BD FACSAria III cell sorter (San Jose, CA, USA) and analyzed using BD FACSDiva Software 7.0 (San Jose, CA, USA). TNF*α* for cell culture was purchased from Life Technologies (#PSC3015, Waltham, MA, USA).

### Lymphoma xenograft in SCID mice

SCID mice were purchased from the Laboratory Animal Center of PEKING University (Beijing, China). Twenty SCID mice at 4 weeks of age were divided into two groups at random. Two million Raji cells were first infected with Ad-GFP and Ad-RCAN1.1-GFP for 48 h before s.c. injection into SCID mice. The body weight and tumor diagrams were recorded every day for 42 days after implantation. The animal studies were performed according to institutional regulations and in facilities approved by the Chinese Council on Animal Care.

### Immunobloting and co-IP analysis

Co-IP and IP were performed as described.^31^ The primary antibodies anti-TBP (T1827) and anti-actin mAb (A2228) were from Sigma-Aldrich (Shanghai, China). Anti-p65 (#4764), anti-I*κ*B*α* (#4814s) and anti-cleaved caspase-3 (#9661) antibody were from Cell Signaling Tech (Danvers, MA, USA). Anti-myc (9E10) was from Abcam (Cat: ab32, Mouse monoclonal, Cambridge, MA, USA). Anti-I*κ*B*α* (phospho-Y42) (Rabbit polyclonal, Cat: BS4736,) and anti-I*κ*B*α* (phospho-S32/S36) (Rabbit polyclonal, Cat: BS4105) were from Bioworld Tech (St. Louis Park, MN, USA). The PageRuler Prestained Protein Ladder from Thermo Scientific (#26616, Waltham, MA, USA) was used to indicate the target protein. Detection and quantification were performed with the Li-cor Odyssey imaging system and its software (Lincoln, NE, USA).

### Dual luciferase assay, nucleus extraction and EMSA

Dual luciferase activity was determined as previously described.^35^ Nuclear extraction was performed using Nuclear Extraction Kit (Millipore, Beijing, China) following the manufacturer's instructions. EMSA was performed using the Odyssey Infrared EMSA Kit (LI-COR Biosciences Co., Lincoln, NE, USA). The consensus sequence for the NF-*κ*B probe is 5′-agttgaggggactttcccaggc-3′.

### Data analysis

All the experiments were repeated three to five times. For immunoblotting, one representative picture is shown; quantifications were from three to five independent experiments. Values represent mean±S.E.M. The data were evaluated for statistical significance with two-way ANOVA or Student's *t*-test.

## Abbreviations

NF-*κ*B: nuclear factor-*κ*B
RCAN1: the regulator of calcineurin 1
BL: Burkitt's lymphoma
CCK8: cell counting kit-8
Co-IP: co-immunoprecipitation
EMSA: electrophoretic mobility shift assay
H_2_O_2_: hydrogen peroxide
I*κ*B*α*: inhibitor of *κ*B
IKK: Inhibition of I*κ*B kinase
NFAT: nuclear factor of activated T-cell
SCID: severe combined immunodeficiency mouse
SYK: spleen tyrosine kinase
TNF-*α*: tumor necrosis factor *α*
Y42: tyrosine 42
